# Comparative anatomy of the felid brachial plexus reflects differing hunting strategies between Pantherinae (snow leopard, *Panthera uncia*) and Felinae (domestic cat, *Felis catus*)

**DOI:** 10.1371/journal.pone.0289660

**Published:** 2023-08-09

**Authors:** Margaret I. Hall, Tyler Lindvall, Ana Suarez-Venot, Dominik Valdez, Heather F. Smith

**Affiliations:** 1 Department of Anatomy, College of Graduate Studies, Midwestern University, Glendale, Arizona, United States of America; 2 Arizona College of Osteopathic Medicine, Midwestern University, Glendale, Arizona, United States of America; 3 College of Veterinary Medicine, Midwestern University, Glendale, Arizona, United States of America; 4 School of Human Evolution and Social Change, Arizona State University, Tempe, Arizona, United States of America; AIIMS: All India Institute of Medical Sciences, INDIA

## Abstract

The brachial plexus, a network of ventral rami providing somatic sensory and motor innervation to the forelimb, is of particular importance in felids. Large-bodied pantherines require powerful rotatory and joint stabilizing forelimb muscles to maintain secure holds on large prey, while smaller-bodied felines are small prey specialists reliant on manual dexterity. Brachial plexus dissections of two snow leopards (*Panthera uncia*) and two domestic cats (*Felis catus*) revealed that generally the morphology of the brachial plexus is quite conserved. However, differences in the nerves supplying the shoulder and antebrachium may reflect differing prey capture strategies between the subfamilies. The brachial plexus of both species derives from ventral rami of C6-T1. In *P*. *uncia*, an extensive musculus (m.) subscapularis with multiple pennations is innervated by a larger number of nn. subscapulares, deriving from more spinal cord levels than in *F*. *catus*. C6 continues to become n. suprascapularis in both taxa; however, in *F*. *catus*, it also gives branches that join with C7, while in *P*. *uncia*, it is dedicated to musculi (mm.) supraspinatus, infraspinatus, and a small branch to cervical musculature. In *F*. *catus*, nervus (n.) medianus receives direct contributions from more ventral rami than *P*. *uncia*, possibly reflecting a greater reliance on manual dexterity in prey capture in the former. In addition to primary innervation by n. thoracodorsalis, m. latissimus dorsi is also innervated by n. thoracicus lateralis near the axilla in both taxa, suggesting that it may belong to a complex of proximal forelimb musculature along with mm. pectoralis profundus and cutaneus trunci.

## Introduction

Carnivoran species can generally be classified as falling into one of two dietary specialization groups, largely determined by body mass [1–3]. Among felids, taxa with body masses of >25 kg will typically focus on hunting large prey, as that hunting strategy is most energy efficient [3]. Most large-bodied felids in this size class belong to the subfamily Pantherinae and have the capability of killing prey more than double their own body mass. Felid species <15 kg hunt exclusively small prey [3]. These species belong to the subfamily Felinae, and primarily hunt prey significantly smaller than themselves. Felids in the intermediate range, 15–25 kg, are variable in hunting strategy. The snow leopard (*Panthera uncia*) and domestic cat (*Felis catus*) exemplify the two different prey capture strategies between the two felid subfamilies, the Pantherinae and Felinae.

*Panthera uncia* hunts a wide array of taxa from small rodents to large Siberian ibex (*Capra sibirica*) [4]. As a solitary hunter it relies on overhead ambush and powerful grasping to immobilize large prey [5]. In contrast, felines such as *F*. *catus* primarily hunt small rodents and birds and typically focus on manual acquisition of prey [2, 6].

Fundamental adaptive differences exist in the forelimb myological patterns in pantherines compared to felines, which reflect their different prey capture strategies [3, 6–8]. To sustain a secure hold while grappling with large prey, pantherines typically require robust joint shoulder musculature and powerful joint-stabilizing forelimb muscles [6, 9]. This pattern holds true in *P*. *uncia*, which displays forelimb musculature suited to hunting behavior intermediate to scansorial and cursorial species, which is required for restraining large prey while remaining flexible enough to climb uneven surfaces [9]. In contrast, as small prey specialists, many felines possess robust digital flexor and extensor muscles, which facilitate manual dexterity [6]. Highly cursorial carnivorans typically have a proportionately larger proximal muscle mass in the forelimb, while those that rely on manual dexterity possess comparatively larger distal forelimb musculature [10, 11].

While previous studies have revealed significant adaptive muscular differences in the forelimbs between pantherines and felines, the neurological mechanisms supporting these differences are still poorly understood. While the brachial plexus has recently been studied in a variety of feline species, including *Leopardus geoffroyi* [12], *Leopardus pardalis* [13], *Puma concolor* [14], *Puma yagouaroundi* [15], and the Van cat (a breed of *Felis catus*) [16], it remains largely unexplored among Pantherinae. This study investigates the morphology of the brachial plexus in representative pantherine and feline species to identify adaptive peripheral nerve differences between these small and large prey specialists.

## Materials and methods

The sample consisted of one pantherine species, *P*. *uncia*, and one feline species, *F*. *catus*. The two *P*. *uncia* specimens included a 22-year-old male (studbook number 2012) and 16-year-old female (studbook number 2438) that each died of natural causes at Santa Barbara Zoological Gardens (SBZ) and were subsequently donated to the Arizona Research Collection for Integrative Vertebrate Education and Study (ARCIVES) at Midwestern University. The specimens were previously dissected for a study of forelimb myology [9]. They were preserved via perfusion into the arterial system with a 10% formaldehyde solution. This research was approved by SBZ and *P*. *uncia* Species Survival Plan (SLSSP). Two male *F*. *catus* specimens were obtained from Carolina Biological Supply Company and were commercially preserved in Carosafe®.

Standard brachial plexus dissections techniques were performed on both specimens. Briefly, the skin was incised in the ventral midline of the trunk and reflected dorsally. Musculi pectorales were identified and reflected. Arteria axillaria was identified and used as a landmark to initially identify the brachial plexus. To identify and confirm the branches of the brachial plexus, both primary literature and standard veterinary atlases were used [9, 16–21]. We use standard anatomical terminology from the Nomina Anatomica Veterinaria, 6^th^ edition [22]. All aspects of the dissections were documented via digital photography using a Canon EOS 60D. The data that support the findings of this study are openly available in this paper.

## Results

**Fig 1:**
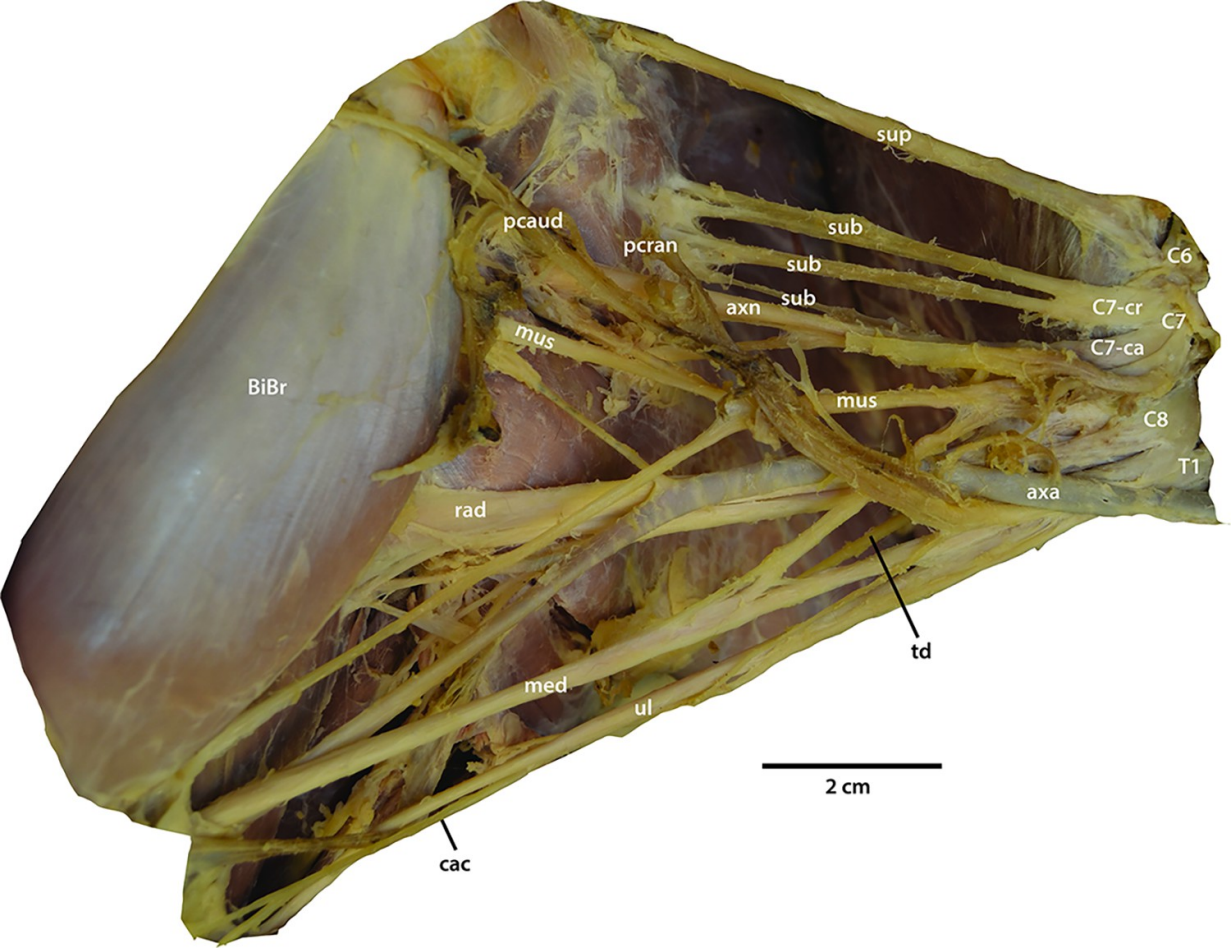
Photograph of the brachial plexus of the pantherine *Panthera uncia*. Abbreviations: axa = a. axillaris; axn = n. axillaris; BiBr = m. biceps brachii; C6, 7, 8 = ventral rami of cervical spinal cord segments 6, 7, and 8; C7-ca = C7 ventral ramus caudal division; C7-cr = C7 ventral ramus cranial division; cac = n. cutaneus antebrachii caudalis; med = n. medianus; mus = n. musculocutaneus; pcran = n. pectoralis cranialis; pcaud = n. pectoralis caudalis; rad = n. radialis; sub = n. subscapularis; sup = n. suprascapularis; T1 = ventral ramus of first thoracic spinal cord segment; td = n. thoracodorsalis; ul = n. ulnaris.

**Fig 2:**
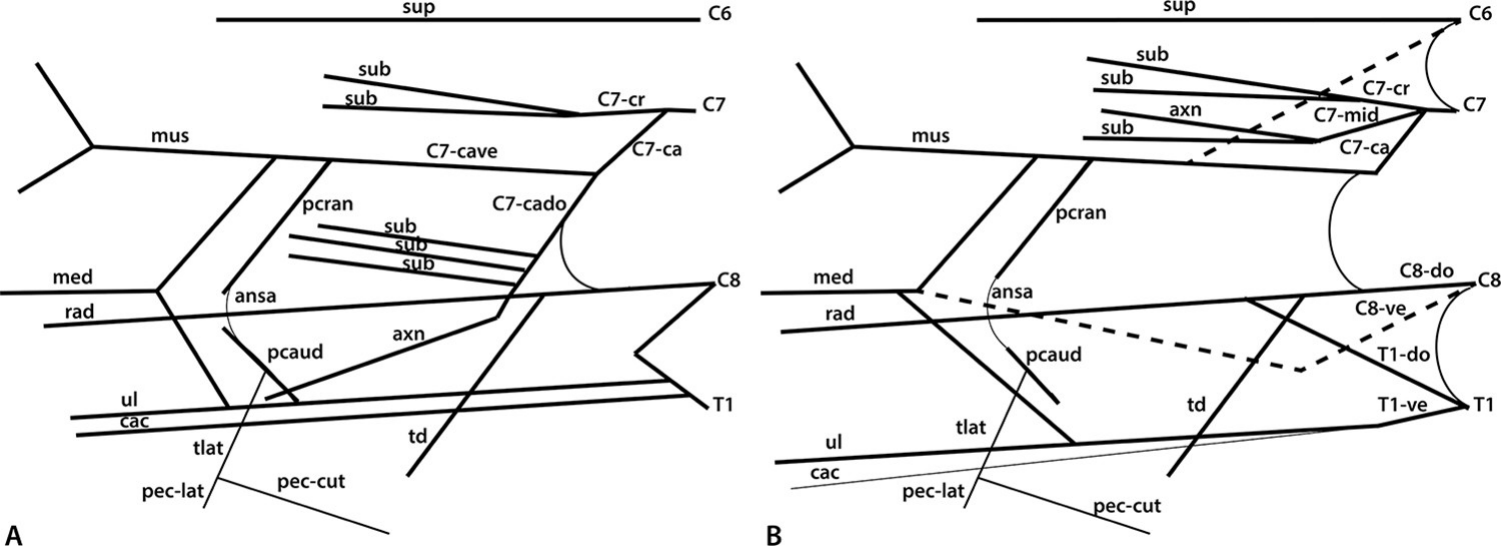
Schematic illustrations of the brachial plexus branching patterns in: A) *Panthera uncia*; B) *Felis catus*. Abbreviations: ansa = ansa pectoralis; axn = n. axillaris; C6, 7, 8 = ventral rami of cervical spinal cord segments 6, 7, and 8; C7-cr = C7 ventral ramus cranial division; C7-ca = C7 ventral ramus caudal division; C7-cado = C7 ventral ramus caudal-dorsal branch; C7-cave = C7 ventral ramus caudal-ventral branch; C7-mid = C7 ventral ramus middle division; C8-do = C8 ventral ramus dorsal division; C8-ve = C8 ventral ramus ventral division; cac = n. cutaneus antebrachii caudalis; med = n. medianus; mus = n. musculocutaneus; pcran = n. pectoralis cranialis; pcaud = n. pectoralis caudalis; pec-lat = pectoral branch to m. latissimus dorsi off n. thoracicus lateralis; pec-cut = pectoral branch to m. cutaneous trunci off n. thoracicus lateralis; rad = n. radialis; sub = n. subscapularis; sup = n. suprascapularis; T1 = ventral ramus of first thoracic spinal cord segment; T1-do = T1 ventral ramus dorsal division; T1-ve = T1 ventral ramus ventral division; tlat = n. thoracicus lateralis; td = n. thoracodorsalis; ul = n. ulnaris.

In both *P*. *uncia* and *F*. *catus*, all the typical nerve branches generally reported in the literature for carnivorans were identified. In both species, the ventral rami of C6, C7, C8 and T1 comprised the brachial plexus, with no contribution from C5 or T2 in any specimen (Fig 1). Within each species, all four dissections presented fundamentally the same pattern; however, there were a few key differences between taxa, discussed in more detail below. Since the *F*. *catus* brachial plexus has been previously described, it is not described in as much detail, and primarily differences with *P*. *uncia* and deviations from previously published patterns are described (Fig 2).

### Panthera uncia

#### Ventral rami- *P*. *uncia*

*C6*. A gracile communication exists between C6 and C7 (Fig 1). However, the C6 ventral ramus continues robustly as n. suprascapularis and grossly does not appear to include any fibers from C7.

*C7*. This robust root divides immediately into a cranial and a caudal branch after possibly receiving a small number of fibers from C6 (Fig 2). The cranial branch divides into two large nn. subscapulares. The caudal branch divides into dorsal and ventral portions, here referred to as “dorsal caudal C7” and “ventral caudal C7” (Fig 2). Dorsal caudal C7 shares fibers laterally with C8 (Figs 1 and 2), and then gives off 3–5 additional smaller nn. subscapulares before continuing as n. axillaris. Ventral caudal C7 initially gives off n. pectoralis cranialis, which has a robust communication to n. pectoralis caudalis (discussed further below). Ventral caudal C7 provides a contribution to n. medianus and then continues laterally as n. musculocutaneus.

*C8*. All branches derived from C8 travel dorsally within the forelimb. C8 communicates laterally with dorsal caudal C7, as discussed above, and medially with T1 (Fig 2). The connection between C8 and T1 is the largest and most robust connection between the roots of the *P*. *uncia* brachial plexus. C8 gives off n. thoracodorsalis and then becomes n. radialis, which immediately subdivides into two branches.

*T1*. After the connection with C8, T1 gives off n. cutaneus antebrachii caudalis, and then n. pectoralis caudalis (Fig 2). After communicating with n. cutaneus antebrachii lateralis, n. pectoralis caudalis emits n. thoracicus lateralis. Lateral to nn. pectorales, T1 contributes to n. medianus and then continues as n. ulnaris, which travels with n. cutaneus antebrachii caudalis to the caudomedial aspect of the elbow.

An “M” is created by nn. musculocutaneus, medianus, and ulnaris, plus the contribution from ventral caudal C7, and a contribution from T1 (Figs 1 and 2). Arteria axillaris travels through the proximal part of the “M” before continuing into the brachium deep to n. medianus. Contributions of ventral rami to peripheral branches are presented in Table 1 and outlined in detail below.

**Table 1:** Peripheral nerves of the brachial plexus, nerve root origins, and muscles innervated in *Panthera uncia* and *Felis catus*.

Peripheral nerve	Taxon	Origin	Motor innervation
Suprascapularis	*P*. *uncia*	C6	Mm. supraspinatus, infraspinatus
	*F*. *catus*	C6-7	Mm. supraspinatus, infraspinatus, brachiocephalicus
Subscapulares	*P*. *uncia*	C7-T1	Mm. subscapularis, pectoralis profundus
	*F*. *catus*	C6-7	Mm. subscapularis, teres major
Pectoralis cranialis	*P*. *uncia*	C7	M. pectoralis superficialis
	*F*. *catus*	C7	M. pectoralis superficialis
Pectoralis caudalis	*P*. *uncia*	C8-T1	M. pectoralis profundus
	*F*. *catus*	C8-T1	M. pectoralis profundus
Thoracicus lateralis	*P*. *uncia*	C8-T1	Mm. latissimus dorsi, cutaneus trunci
	*F*. *catus*	C8-T1	Mm. latissimus dorsi, cutaneus trunci
Thoracodorsalis	*P*. *uncia*	C7-T1	M. latissimus dorsi
	*F*. *catus*	C7-8	M. latissimus dorsi
Axillaris	*P*. *uncia*	C7-T1	Mm. deltoideus, teres minor, teres major
	*F*. *catus*	C6-7	Mm. deltoideus, teres minor
Musculocutaneus	*P*. *uncia*	C7	Mm. articularis humeri, biceps brachii, brachialis
	*F*. *catus*	C6-8	Mm. articularis humeri, biceps brachii, brachialis
Medianus	*P*. *uncia*	C7-T1	Caudal antebrachial musculature
	*F*. *catus*	C6-T1	Caudal antebrachial musculature
Ulnaris	*P*. *uncia*	C8-T1	Mm. flexor carpi ulnaris and flexor digitorum profundus caput ulnare
	*F*. *catus*	C8-T1	Mm. flexor carpi ulnaris and flexor digitorum profundus caput ulnare
Radialis	*P*. *uncia*	C7-T1	Mm. triceps brachii complex, tensor fascia antebrachii, antebrachial extensor compartment
	*F*. *catus*	C7-T1	Mm. triceps brachii complex, antebrachial extensor compartment

#### Terminal branches- *P*. *uncia*

*N*. *suprascapularis*. The ventral ramus of C6 continues laterally to become nervus (n.) suprascapularis (Figs 1 and 2). It dives deep between the bellies of musculi (mm.) subscapularis and supraspinatus to continue to the dorsal side of the scapula, first innervating musculus (m.) supraspinatus and then wrapping around the scapular spine to innervate m. infraspinatus.

*Nn*. *subscapularis*. Three to five distinct nn. subscapulares are present in *P*. *uncia* (Figs 1 and 2). The two most proximal nn. subscapulares are consistently large branches directly off the cranial division of C7. An additional 3–5 smaller nn. subscapulares branch off the dorsal caudal division of C7. All nn. subscapulares dive directly into the multipennate muscle tissue of m. subscapularis.

*Nn*. *Pectorales*. Nervus pectoralis cranialis is a proximal branch off the ventral caudal division of C7 (Figs 1 and 2). Nervus pectoralis caudalis is a branch of T1 after the latter gives off n. cutaneus antebrachii medialis. The two nn. pectorales communicate robustly with each other in an arc, the ansa pectoralis, before innervating the extensive pectoral musculature. Nervus pectoralis caudalis then gives off n. thoracicus lateralis.

*N*. *thoracicus lateralis*. This nerve emerges as a small branch off n. pectoralis caudalis (Fig 2). Since the *P*. *uncia* study specimens were already dissected for a previous study [9], m. cutaneus trunci and mm. pectoralis had already been removed. However, the branch from n. thoracicus lateralis to m. latissimus dorsi was retained (Fig 1).

*N*. *thoracodorsalis*. Nervus thoracodorsalis is a branch off the ventral ramus of C8 (Figs 1 and 2). It travels directly to m. latissimus dorsi. However, it is not the only nerve to innervate m. latissimus dorsi, as n. thoracicus lateralis also travels there.

*N*. *axillaris*. After the dorsal caudal division of C7 gives off nn. subscapulares, it continues as n. axillaris (Figs 1 and 2). The nerve courses laterally before dividing into two branches. The larger branch dives deep into the space between mm. subscapularis, teres major, and articularis humeri with arteria (a.) circumflexa humeri caudalis, and reemerges in the quadrangular space. The smaller branch dives proximally into the space between mm. teres major and subscapularis.

*N*. *musculocutaneus*. After giving off n. pectoralis cranialis, the ventral caudal division of C7 splits into n. musculocutaneus and a contribution to n. medianus (Figs 1 and 2). Nervus musculocutaneus bifurcates into a large branch that dives into the brachial musculature, and a smaller branch that courses distally towards the cubital fossa. The large branch travels along the deep surface of m. biceps brachii through the brachium and emits deep rami musculares to m. brachialis. After giving off its muscular branches, it terminates as n. cutaneus antebrachii medialis.

*N*. *medianus*. Nervus medianus is formed by contributions from ventral caudal C7 and T1 (Figs 1 and 2). The nerve courses in a straight path through the brachium without giving off any branches. It courses through foramen supracondylare of the humerus to enter the cubital fossa. In the antebrachium, it gives off muscular branches to the caudal antebrachial musculature.

*N*. *ulnaris*. After T1 gives off its contribution to n. medianus, it continues as n. ulnaris (Figs 1 and 2 and courses straight towards the medial elbow. It gives off n. cutaneus antebrachii caudalis, which diverges to travel superficially before coursing around medialis epicondylus and diving deep to m. epitrochleoanconeus. It then courses through the antebrachium deep to m. flexor carpi ulnaris.

*N*. *radialis*. After C8 gives off n. thoracodorsalis, it continues as n. radialis (Figs 1 and 2). It courses deep to a. axillaris through the axilla where it subdivides, sending rami musculares to mm. tensor fascia antebrachii and triceps brachii. It emerges from m. triceps brachii pars medialis to course distolaterally past m. brachioradialis. In one *P*. *uncia* specimen, it also gave off a small muscular branch to m. brachialis. A small branch, n. cutaneus antebrachii lateralis, courses superficially toward the skin. The remainder of the nerve courses distally and dives deep into the belly of m. extensor carpi radialis longus. It gives off rami musculares to the remaining cranial antebrachial musculature.

### Felis catus brachial plexus

#### Ventral rami- *F*. *catus*

**Fig 3:**
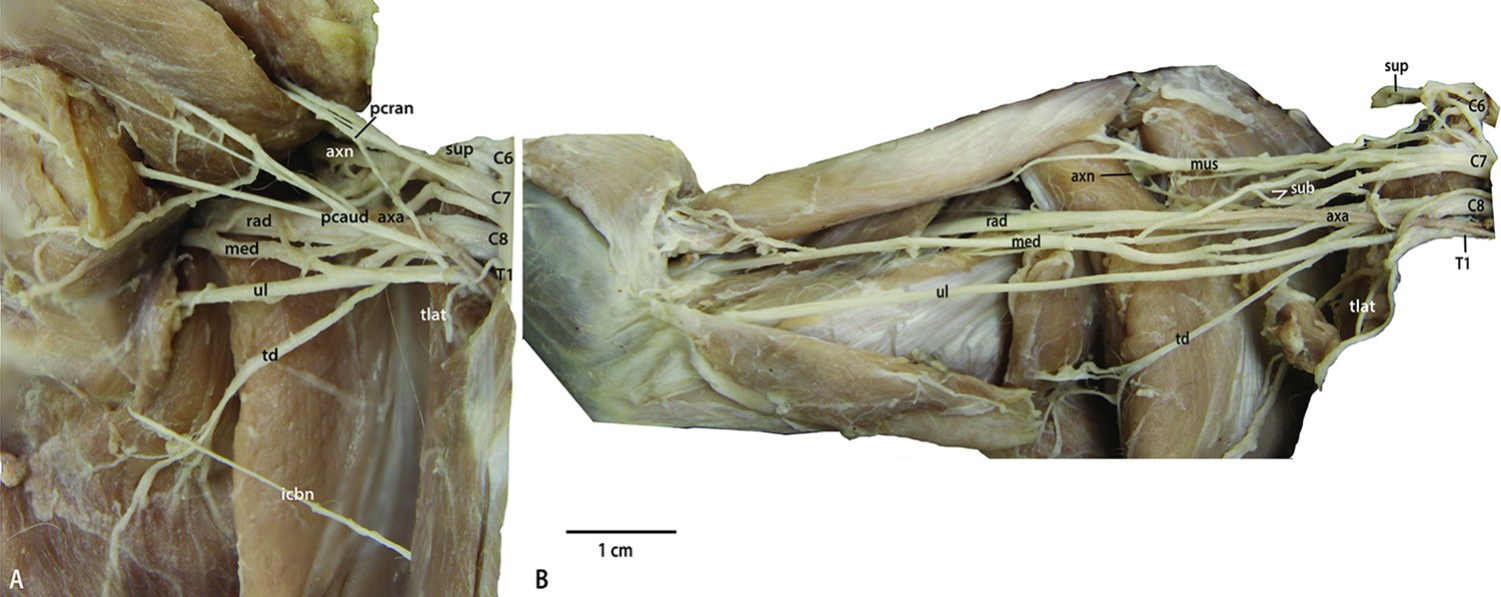
Photographs of the brachial plexus of the feline *Felis catus*: A) Superficial view showing relationship with pectoral muscles; B) Deep view revealing deeper nerve branches. Abbreviations: axa = a. axillaris; axn = n. axillaris; C6, 7, 8 = ventral rami of cervical spinal cord segments 6, 7, and 8; icbn = n. intercostobrachialis; med = n. medianus; mus = n. musculocutaneus; pcran = n. pectoralis cranialis; pcaud = n. pectoralis caudalis; rad = n. radialis; sub = n. subscapularis; sup = n. suprascapularis; T1 = ventral ramus of first thoracic spinal cord segment; td = n. thoracodorsalis; tlat = n. thoracicus lateralis; ul = n. ulnaris.

*C6*. From proximal to distal, the root of C6 provides contributions to n. phrenicus, exchanges a communication with the cranial branch of C7 (see below), and sends a communicating branch to n. musculocutaneus before becoming a large n. suprascapularis (Figs 2 and 3).

*C7*. This large root immediately divides into three branches, herein named “cranial,” “middle,” and “caudal”. The cranial and middle branches create dorsal division nerves, while the caudal branch gives rise to both ventral and dorsal nerves (Fig 2). The cranial branch communicates with C6 (above) and then divides into two distinct nn. subscapulares. The middle branch of C7 divides proximally into n. axillaris and another n. subscapularis. The caudal branch contributes to n. thoracicus longus, emits n. pectoralis cranialis, contributes to n. medianus, and then becomes n. musculocutaneus. The caudal branch of C7 also has a dorsal portion that contributes to n. radialis, and a second independent communication contributing to n. thoracodorsalis (see below).

*C8*. Both dorsal and ventral branches extend from C8. The ventral branch communicates with both C7 and T1, and then forms n. medianus (Fig 2). However, while these connections are common in mammalian brachial plexuses, the specimens dissected in this study do not exhibit the typical “M” pattern. The dorsal division of C8 communicates with T1 and the caudal branch of C7, but it extends n. thoracodorsalis before communicating with T1. A second branch from the caudal branch of C7 communicates directly with n. thoracodorsalis. After communicating with T1, the dorsal branch of C8 becomes n. radialis. The ventral branch of C8 gives off n. pectoralis caudalis.

*T1*. T1 also divides immediately into dorsal and ventral branches (Fig 2). The dorsal branch contributes to n. radialis. The ventral branch communicates with the accessory branch to m. latissimus dorsi from m. thoracicus lateralis. The ventral branch contributes to n. medianus before continuing to the antebrachium as n. ulnaris.

#### Terminal branches- *F*. *catus*

*N*. *suprascapularis*. Nervus suprascapularis is the distal continuation of the ventral ramus of C6 after it emits its other branches (Figs 2 and 3). It shares a common trunk with a cranially directed branch that travels towards m. brachiocephalicus.

*Nn*. *subscapularis*. Two nervi subscapulares were identified branching off the cranial branch of C7 and coursing directly to m. subscapularis (Fig 2). A third n. subscapularis branches off the middle branch of C7, which dives into m. teres major.

*Nn*. *Pectorales*. The nervi pectorales originate from different ventral rami but share a connection, the ansa pectoralis. Nervus pectoralis cranialis emerges as a branch off the caudal branch of C7. It courses caudally to join n. pectoralis caudalis, which branches off the ventral branch of C8. The nn. pectorales innervate mm. pectorales and give off n. thoracicus lateralis.

*N*. *thoracicus lateralis*. Nervus thoracicus lateralis emerges as a branch from n. pectoralis caudalis (Fig 2). It sends a supplementary branch to m. latissimus dorsi before continuing to m. cutaneus trunci.

*N*. *thoracodorsalis*. This nerve is a branch of the dorsal branch of C8 distal to its connection to C7 (Fig 2). It courses directly to m. latissimus dorsi and dives into the muscle belly.

*N*. *axillaris*. Nervus axillaris is a terminal branch of the middle branch of C7, along with the third n. subscapularis (Fig 2). It courses deep into the axilla, similar to the pattern in *P*. *uncia*.

*N*. *musculocutaneus*. Nervus musculocutaneus is the distal continuation of the caudal branch of C7 after it gives off its other contributions (Figs 2 and 3). It splits into proximal and distal branches as it courses into the brachium, as in *P*. *uncia*.

*N*. *medianus*. Nervus medianus in *F*. *catus* is formed by contributions from the caudal branch of C7, ventral branch of C8, and ventral branch of T1 (Figs 2 and 3). Its distal course is similar to that of *P*. *uncia*.

*N*. *ulnaris*. The ventral branch of T1 becomes n. ulnaris after giving off its contributions to n. medianus (Figs 2 and 3). Its course is similar to that of *P*. *uncia*.

*N*. *radialis*. Nervus radialis is the continuation of the dorsal branch of C8 (Figs 2 and 3). It also receives contributions from the dorsal caudal branch of C7 and the caudal branch of T1. Its course is similar to that of *P*. *uncia*.

## Discussion

### Functional anatomical differences between the brachial plexus of felines and pantherines

The brachial plexus of *P*. *uncia* (subfamily Pantherinae) and *F*. *catus* (subfamily Felinae) were found to be largely similar to each other and to the few other documented feline taxa (for a recent review, see [15]). However, several notable differences appear to reflect the differing prey capture strategies between large-bodied pantherines and smaller felines. In particular, pantherines tend to rely on their enlarged scapular musculature [9, 23] to subdue prey that are larger than themselves, and this emphasis is reflected in the brachial plexus branches supplying the scapular musculature. In contrast, felines emphasize flexion of the digits, which is reflected in additional spinal cord levels contributing directly to the nerves that supply the digital and carpal flexors.

The composition of nn. subscapulares exhibit a noteworthy difference between *P*. *uncia* and felines. In felids, typically an extremely large C7 contributes to many peripheral branches of the brachial plexus [15]. However, in *P*. *uncia*, two distinct nn. subscapulares are created by the cranial branch of C7, plus three additional nn. subscapulares derive from the caudal dorsal branch of C7 distal to its connection with C8. This arrangement indicates that C8 (and possibly T1) fibers are also present in the more caudally positioned nn. subscapulares in *P*. *uncia*, a contribution not noted in previous felid studies [e.g., 15]. As in *P*. *uncia*, the cranial branch of C7 in *Felis catus* divides into two distinct nn. subscapulares. However, the middle branch of C7 divides into n. axillaris and a third, caudally positioned n. subscapularis, that dives directly into m. teres major, rather than the five individual subscapular nerves observed in *P*. *uncia*. The pattern in *F*. *catus* mirrors that of other published felines in which 2–3 nn. subscapulares are typically present, such as *L*. *geoffroyi* [12], *L*. *pardalis* [13], *P*. *concolor* [14], *P*. *yagouaroundi* [15], and the Van cat [16].

**Fig 4:**
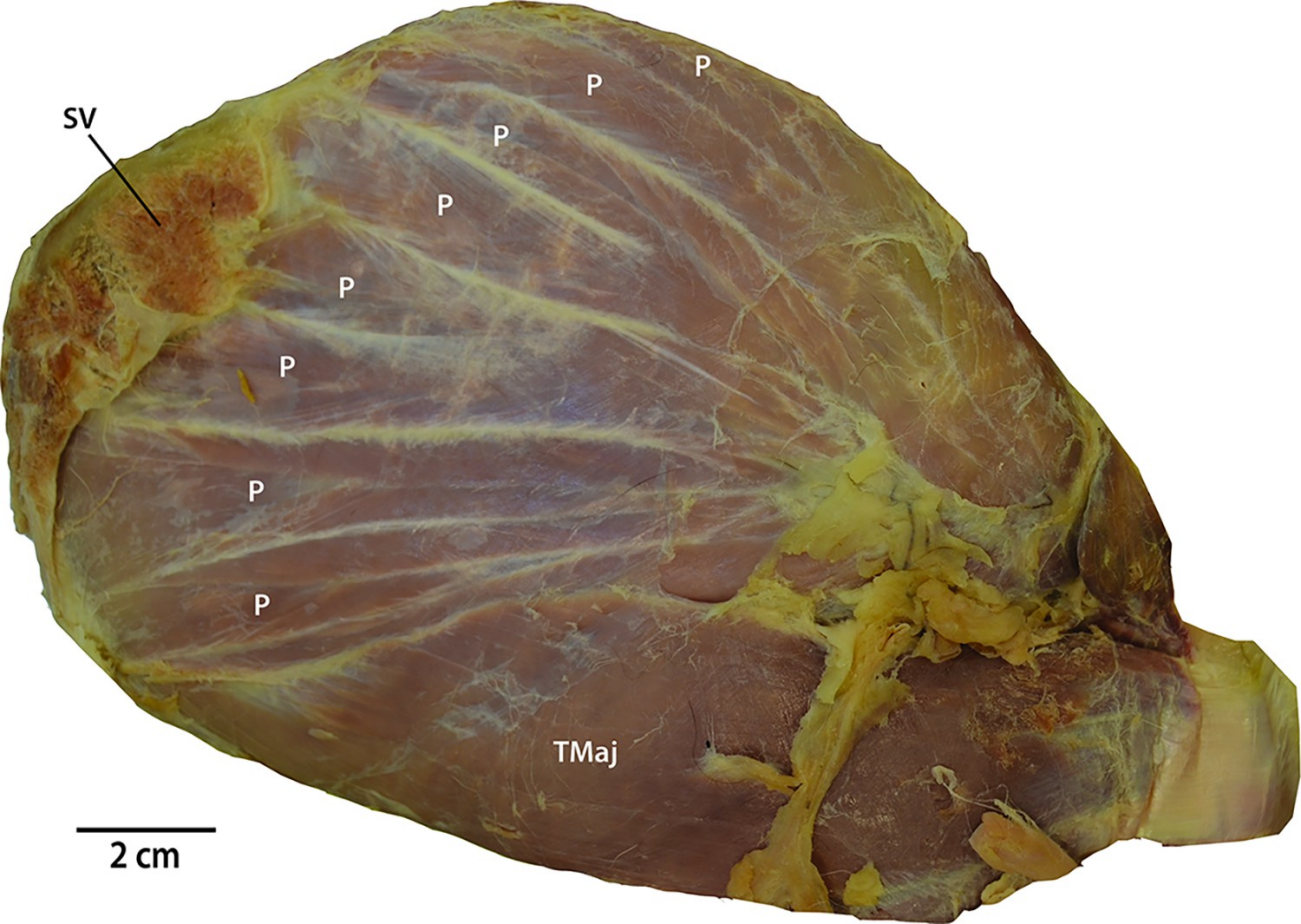
Digital photograph of the medial surface of the left scapula of *Panthera uncia* demonstrating the multiple pennations of m. subscapularis. Abbreviations: P = muscular pennation; SV = m. serratus ventralis; TMaj: m. teres major.

We suggest that the number of nn. subscapulares in each species likely relates both to size and scapular muscle morphology. In *P*. *uncia*, m. subscapularis is proportionately massive and displays 8–9 large, individually identifiable pennations [9] (Fig 4). These pennations may require more extensive innervation than the simple single-bellied m. subscapularis of *F*. *catus*. Interestingly, *P*. *concolor*, a larger-bodied member of the Felinae subfamily, also exhibits several individual nn. subscapulares, the first exclusively from C6, the second from C6 and C7, and the third and fourth from only C7; however, no contributions from C8 or T1 have been described [14]. In the medium-sized feline *P*. *yagouaroundi*, there are two nn. subscapulares which derive from a common trunk off a branch of C6-7 [15]. The nervi subscapulares are composed of C6-7 fibers in most documented felines, including *F*. *catus* [18], *L*. *geoffroyi* [12], *L*. *pardalis* [13], *P*. *concolor* [14], *P*. *yagouaroundi* [15], and the Van cat [16]. Future studies including additional pantherine taxa could reveal whether the patterns observed here are consistently different between felines and pantherines.

*Panthera uncia* has only a very gracile connection between the ventral rami of C6 and C7, and from gross dissection it appears that they share relatively few fibers. However, in our *F*. *catus* specimens, C6 and C7 shared two robust connections, both medially between the roots of C6 and C7 and laterally as the root of C6 sends a branch to n. musculocutaneus, a communication not present in *P*. *uncia* nor noted in previous felid studies [12–18]. This difference suggests a pattern of greater integration and overlapping nerve territories of the C7 branches in *F*. *catus*. Similarly, C6 contributes to other peripheral nerves such as n. axillaris and musculocutaneous in other felines, including *L*. *geoffroyi* [12], *P*. *concolor* [14], *P*. *yagouaroundi* [15], and the Van cat [16]. In contrast, in *P*. *uncia*, the sole function of C6 appears to be dedicated innervation of the snow leopard’s extensive shoulder musculature via n. suprascapularis.

In *F*. *catus* and other felines, n. medianus receives direct, robust contributions from C7, C8, and T1. In *F*. *catus*, it is a large nerve that supplies the domestic cat’s well-developed digital flexor muscles. In contrast, in *P*. *uncia*, n. medianus is composed of direct branches from only C7 and T1, although a proximal connection between C8 and T1 suggests some C8 fibers may also be included. The direct contributions from a larger number of spinal cord levels in felines may reflect the increased emphasis on fine manual manipulation.

Both *P*. *uncia* and *F*. *catus*, as well as previously documented felines [15], demonstrate significant connections between C8 and T1. In *F*. *catus*, T1 divides into a dorsal portion that sends a large communication to n. radialis, and a ventral portion that becomes n. ulnaris. However, in *P*. *uncia* any dorsally directed contribution from T1 to n. radialis occurs in the medial connection, without further branches from T1 to n. radialis. In both species, the continuation of T1 becomes a large n. ulnaris, but in *P*. *uncia* an exceptionally large n. cutaneus antebrachii caudalis also emanates from the root of T1. This nerve is significantly more gracile in *F*. *catus*. While the reason for a difference in cutaneous sensory needs between the taxa is not immediately apparent, we suggest that difficulty grappling with prey may cause *P*. *uncia* to require more extensive caudal cutaneous innervation of the antebrachium. An alternative interpretation is that the robust peripheral nerves in *P*. *uncia* may be an adaptation to extremely cold temperatures or simply an allometric effect of its larger body size.

### A muscular sling of the forelimb

We hypothesize, at least in carnivorans, since mm cutaneous trunci, pectoralis profundus, and latissimus dorsi display common innervation, that ancestrally they may have derived from a laterally positioned, common muscular sling. While ventral rami provide all the component nerves to the brachial plexus, many researchers have found it useful to compartmentalize these nerves into dorsal and ventral divisions to indicate which nerves innervate muscles that are developmentally dorsal or ventral to the humerus [24], as we have here. However, the present study has revealed that the position and targets of n. thoracicus lateralis unite muscles that are developmentally and positionally both dorsal and ventral. In both *F*. *catus* and *P*. *uncia*, n. thoracicus lateralis extends from n. pectoralis caudalis, which primarily innervates the ventrally positioned m. pectoralis profundus and pierces m. pectoralis superficialis. Nervus thoracicus lateralis of *F*. *catus* also extends laterally and dorsally to send individual branches to m. latissimus dorsi, a developmentally dorsal muscle, and to m. cutaneus trunci. The latter is not generally considered to be a muscle of the forelimb, although this has been a topic of discussion in the literature [25–27].

In *P*. *uncia*, due to previous dissections of the specimens, we were only able to identify the origin of n. thoracicus lateralis from n. pectoralis caudalis and a branch from n. thoracicus lateralis innervating m. latissimus dorsi. Thus, the further distribution of n. thoracicus lateralis and of n. pectoralis caudalis could not be identified, although appeared to include fibers from C8 and T1. It has been previously noted that n. thoracicus lateralis of *P*. *yagouaroundi* is also formed by C8 and T1 and innervates mm. cutaneus trunci and pectoralis profundus, but not m. latissimus dorsi [15]. In *P*. *concolor*, n. thoracicus lateralis first emits a branch to m. latissimus dorsi before continuing to m. cutaneus trunci [14], and n. thoracicus lateralis in *L*. *geoffroyi* also innervates mm. cutaneus trunci and latissimus dorsi, but not m. pectorales profundus [12]. In several canid species, n. thoracicus lateralis also innervates both the dorsally positioned m. latissimus dorsi and the ventral aspect of m. pectorales profundus, in addition to m. cutaneus trunci, including in the domestic dog [19] and pampas fox, *Lycalopex gymnocercus* [28]. In other carnivorans, n. thoracicus lateralis innervates only mm. pectorales profundus and cutaneus trunci, as in the European pine marten and red fox [29].

Mm. latissimus dorsi, cutaneous trunci, and pectoralis profundus are linked anatomically, via their insertion points and shared common innervation. In addition, mm. latissimus dorsi and pectoralis profundus share a common function during locomotion, to pull the body anteriorly while the forelimb is stable. In modern carnivorans, m. cutaneous trunci inserts on the humerus near the insertions of the two aforementioned muscles, suggesting that the three may at one time have been functionally linked. Future studies could test this functional-developmental hypothesis by evaluating the innervation patterns of other, non-carnivoran quadrupeds.
